# Discovery and Characterization of an Endo-1,3-Fucanase From Marine Bacterium *Wenyingzhuangia fucanilytica*: A Novel Glycoside Hydrolase Family

**DOI:** 10.3389/fmicb.2020.01674

**Published:** 2020-07-28

**Authors:** Jingjing Shen, Yaoguang Chang, Yuying Zhang, Xuanwei Mei, Changhu Xue

**Affiliations:** ^1^College of Food Science and Engineering, Ocean University of China, Qingdao, China; ^2^Laboratory for Marine Drugs and Bioproducts, Qingdao National Laboratory for Marine Science and Technology, Qingdao, China

**Keywords:** marine bacterium, sulfated fucan, endo-1, 3-fucanase, transcriptomics, UPSEC-MS, oligosaccharide

## Abstract

Sulfated fucans are important marine polysaccharides widely distributed in brown algae and echinoderms, which gained increasing research interest for their various biological and biomedical activities. Fucanases could serve as tools in the bioconversion and structural investigation of sulfated fucans. A few gene-defined endo-1,4-fucanases have been characterized, while the sequence of endo-1,3-fucanase remain unstudied. Here, an endo-1,3-fucanase gene *funA* was screened from the genome of marine bacterium *Wenyingzhuangia fucanilytica* CZ1127^T^ using transcriptomics. None of the previously reported glycoside hydrolase domains were predicted in the enzyme FunA, which hydrolyzed sulfated fucans in a random endo-acting manner. Ultrahigh performance size exclusion chromatography-mass spectrometry and nuclear magnetic resonance analyses revealed that FunA specifically cleaves α-1,3 glycosidic linkage between 2-O-sulfated and non-sulfated fucose residues. FunA exhibited transglycosylating activity with glycerin, methanol, and L-fucose as acceptors. D206 and E264 were critical for the functioning of FunA as identified by the site-directed mutagenesis. Another five homologs of FunA were confirmed to possess endo-1,3-fucanase activities. This is the first report on the sequence of endo-1,3-fucanase. The novelty of FunA and its homologs in sequences and activity shed light on a novel glycoside hydrolase family, GH168.

## Introduction

Sulfated fucans, also known as fucoidans, are sulfated polysaccharides mainly composed of L-fucose and sulfate groups usually extracted from brown algae and echinoderms. Because of their various biological and biomedical characteristics such as being anticoagulant (Colliec et al., 1991; Cumashi et al., 2007), antithrombotic (Min et al., 2012), anti-inflammatory (Cumashi et al., 2007), anticancer (Nagamine et al., 2020), and immunomodulatory (Amin et al., 2020), research interest in sulfated fucans have increased. Additionally, they had been utilized for dermal burn healing (Sezer et al., 2007), wound healing (Murakami et al., 2010) and bone tissue regeneration (Jin and Kim, 2011) due to their excellent biocompatibility. According to the linkage pattern of their backbone, sulfated fucans could be generally classified into two groups (Cumashi et al., 2007): (i) type I sulfated fucans composed of homogeneous 1,3-α-L-fucopyranose residues. They distributed in most of Laminariales and Ectocarpales algae (Schultz-Johansen et al., 2018), and in echinoderms including sea cucumbers (Chen et al., 2012; Yu et al., 2014a, b, 2015; Chang et al., 2016) and sea urchins (Mulloy et al., 1994; Vilela-Silva et al., 1999); (ii) type II sulfated fucans consisting of alternating 1,3- and 1,4-linked α-L-fucopyranose residues. Most of the sulfated fucans from Fucales algae adopted this linkage pattern (Jiao et al., 2011; Schultz-Johansen et al., 2018).

Fucanase is a class of enzyme that specifically catalyzes the hydrolysis of glycoside bonds within sulfated fucans in an endo-acting manner. Fucanases are desirable tools to identify the structure of sulfated fucans (Berteau and Mulloy, 2003) and establish structure-activity relationships (Kusaykin et al., 2016). To date, a few genes of endo-1,4-fucanases (i.e., enzymes cleaving α-1,4 glycosidic linkages in type II sulfated fucan backbones) have been characterized, including FcnA, FFA1, FFA2, Fp273, Fp277, Fp279, P5AFcnA, and P19DFcnA (Colin et al., 2006; Silchenko et al., 2017, 2018; Schultz-Johansen et al., 2018; Vickers et al., 2018). All of the reported endo-1,4-fucanases belong to the glycoside hydrolase (GH) 107 family in the CAZy database^1^. However, the gene for endo-1,3-fucanase, which cleaves α-1,3 glycosidic linkage in type I or type II sulfated fucans in an endo-acting manner are yet to be identified (Schultz-Johansen et al., 2018).

Marine microbes play a crucial role in biogeochemical cycling (Falkowski et al., 2008). The bacteria of family Flavobacteriaceae are generally considered as specialists for the polysaccharide degradation through the various activities of carbohydrate active enzymes (Zhang H. et al., 2019). In our previous work, we isolated a marine bacterium *Wenyingzhuangia fucanilytica* CZ1127^T^ (= CCTCC AB 2015089T = KCTC 42864T) (Chang et al., 2010; Chen et al., 2016), which belongs to family Flavobacteriaceae. Catabolic profiling confirmed this bacterium was versatile in polysaccharide bioconversions, and the polysaccharides degradation was usually related to dedicated genes (Barbeyron et al., 2016). Hence, the sequencing of entire genome was completed recently (GenBank accession no. CP014224). Initial studies indicated that *W. fucanilytica* secreted fucanase using sulfated fucan as the sole carbon source. Utilizing the intracellular enzymes produced by *W. fucanilytica*, we prepared a series of sulfated fucan oligosaccharides composed of 1,3-α-L-fucopyranose, and clarified the structure of several type I sulfated fucans from sea cucumbers including *Acaudina molpadioides* (Yu et al., 2014a), *Apostichopus japonicus* (Yu et al., 2015), *Holothuria tubulosa* (Chang et al., 2016), and *Thelenota ananas* (Yu et al., 2014b). Results of the said study showed that *W. fucanilytica* was capable of producing endo-1,3-fucanase.

For this study then, we aim to determine the sequence coding for endo-1,3-fucanase from *W. fucanilytica* CZ1127^T^. Since there was no endo-1,3-fucanase gene described yet, we screened candidate genes from those that were up-regulated when the *W. fucanilytica* was exposed to sulfated fucans. The candidate genes were cloned and heterologously expressed in *Escherichia coli*, and the enzyme activity, biochemical characteristics, and hydrolysis patterns were subsequently investigated. The novel enzyme described in this study may promote the application of endo-1,3-fucanase in bio-industry.

## Materials and Methods

### Materials

Dried sea cucumber *Isostichopus badionotus* was purchased from a local market in Qingdao (China). The sulfated fucan was extracted following a previously reported method (Chang et al., 2010). Extracts were further purified using an ÄKTA^TM^ Prime Plus system (GE healthcare, Uppsala, Sweden) equipped with a HiPrep^TM^ 26/60 Sephacryl S-400 column (GE Healthcare, Uppsala, Sweden). The purified polysaccharide was dialyzed, lyophilized and used for other downstream processing.

Sulfated fucans from the brown algae *Fucus vesiculosus* and *Macrocystis pyrifera* were purchased from Sigma-Aldrich (St. Louis, MO, United States); sulfated fucan from *Ascophyllum nodosum* was purchased from Carbosynth (Suzhou, China). Reagents used in ultrahigh performance size exclusion chromatography (UPSEC) - mass spectrometry (MS) (UPSEC-MS) were LC-MS grade. D_2_O (99.8%, Sigma-Aldrich, St. Louis, MO, United States) was used in the nuclear magnetic resonance (NMR) experiment. The other reagents were all analytical grade.

### mRNA-Seq Expression Profiling

For transcriptome profiling, the bacterium was cultivated in the minimal medium (Ensor et al., 1999) containing 0.2% (w:v) D-glucose (Sigma-Aldrich, St. Louis, MO, United States) or sulfated fucan from *Isostichopus badionotus*, and incubated overnight at 25°C until it reached midlogarithmic growth phase. Total RNA of *W. fucanilytica* CZ1127^T^ was extracted using RNAprep pure Cell/Bacteria Kit (TIANGEN, Beijing, China). The concentration and quality of RNA were measured using a NanoDrop 2000 Spectrophotometer (Thermo Scientific, Waltham, MA, United States). The RNA integrity was validated via Agilent 2100 Bioanalyzer (Agilent Technologies, United States). The mRNA was enriched using the Illumina Ribo-Zero rRNA Removal Kit (Bacteria) and split into small-fragment after adding the fragmentation buffer. Di-tagged cDNAs were synthesized by successive random priming with terminal-tagging oligos and purified with the AMPure XP beads (Beckman Coulter, Beverly, MA, United States). The enrichment of cDNAs was amplified and then the final mRNA-seq library was generated. Sequencing was then carried out on the Illumina X-Ten platform in Novogene Co., Ltd. (Beijing, China).

Read mapping was performed on the *W. fucanilytica* CZ1127^T^ reference genome (RefSeq NZ_CP014224.1) by Bowtie v2.0.6 and TopHat v2.0.7 software. The number of reads mapping to each predicted coding sequence was counted by HTSeq v0.6.1. Normalization for differential expression analysis and statistical tests were performed using DEGSeq R package v1.12.0 software, and a Bonferroni *p*-value adjustment was used to correct for multiple testing. Genes with an adjusted *p*-value < 0.05 were considered as differentially expressed.

### Gene Cloning and Protein Expression

The genes from the up-regulated genes of *W. fucanilytica* induced by sulfated fucan were selected to be cloned and heterologously expressed in *E. coli*. Genomic DNA of *W. fucanilytica* CZ1127^T^ was extracted using the TIANamp Bacteria DNA Kit (TIANGEN, Beijing, China). The presence of signal peptide was predicted using the SignalP 5.0 server (Armenteros et al., 2019). The genes without predicted signal sequences were amplified by PCR using the forward and reverse primer pair (listed in Supplementary Table S1). Purified PCR fragments were subsequently digested using *Bam*HI/*Xho*I and inserted into the pET 28a(+) vector (Novagen, San Diego, CA, United States) containing a (His)_6_-tag at the N-terminus. The obtained expression vectors were transformed into *E. coli* BL21 (DE3) cells. The transformed cells were then selected by using the Luria-Bertani (LB) agar plates containing 30 μg/mL kanamycin, and cultured in the LB broth at 37°C until OD_600_ reached 0.4. After incubation with 0.5 mM isopropyl β-D-1-thiogalactopyranoside at 17°C for 12 h, cells were collected and disrupted by sonication in 20 mM NaH_2_PO_4_-Na_2_HPO_4_ buffer (pH 8.0).

To purify the expressed protein, the supernatants of the cell lysate were loaded onto the HisTrap HP columns (GE Healthcare, Uppsala, Sweden) and eluted in a linear gradient of 0-0.5 M imidazole in 20 mM NaH_2_PO_4_-Na_2_HPO_4_ buffer (pH 8.0) with 0.3 M NaCl at 4°C. The active fractions were combined and desalted using HiPrep^TM^ 26/10 Desalting column (GE Healthcare, Uppsala, Sweden) against 20 mM NaH_2_PO_4_-Na_2_HPO_4_ buffer (pH 8.0). The purity of the obtained protein was evaluated by SDS-PAGE with a 5% stacking gel and a 12% running gel. Protein bands were visualized by Coomassie brilliant blue (Sigma-Aldrich, St. Louis, MO, United States). The *M*_*w*_ of the enzyme was estimated by comparing with the standard markers (Page Rulerprestained protein ladder, Fermentas, Waltham, MA, United States). The purified enzyme was used in the succeeding experiments.

### Enzymatic Activity Assay

The enzymatic activity was determined by incubating the purified enzyme with 1 mg/mL substrate in 20 mM NaH_2_PO_4_-Na_2_HPO_4_ buffer (pH 8.0) at 40°C for 10 min. The released reduced sugar was quantified by a *p*-hydroxybenzoic acid hydrazide method (Lever, 1972). One unit of activity was defined as the amount of enzyme that released 1.0 μmol reduced sugar (equivalent to L-fucose) per minute. The protein concentration was determined using a BCA Protein Assay Kit (Beyotime Biotechnology, Shanghai, China) with bovine serum albumin as the standard.

### Investigation on Hydrolysis Patterns

To investigate the hydrolysis patterns of the recombinant enzyme, 1 U enzyme solution was incubated with 50 mL sulfated fucan from *Isostichopus badionotus* (Ib-FUC) solution (2 mg/mL, pH 8.0) at 25°C for 12 h. Aliquots of the resulting reaction products were withdrawn at intervals and heated at 100°C for 10 min to deactivate the enzyme. After 12 h reaction, 1 U enzyme was supplemented, and the incubation was continued for another 12 h to obtain the end product. The products were analyzed using high-performance size exclusion chromatography coupled with a refractive index detector (HPSEC-RID) (Agilent 1260, Agilent Technologies, Santa Cruz, CA, United States). A TSKgel SuperAW4000 column (Tosoh Corporation, Kanagawa, Japan) (0.2 M NaCl as the eluent, flow rate 0.6 mL/min) and a Superdex peptide 10/300 GL column (GE healthcare, Uppsala, Sweden) (50 mM ammonium formate as the eluent, flow rate 0.5 mL/min) were, respectively, utilized to examine the global profile and oligosaccharide pattern.

### Biochemical Characteristics and Kinetic Constants

The effect of temperature on the activity was tested by incubating the enzyme with Ib-FUC at 15–60°C, and thermal stability was evaluated by measuring the residual activity of enzyme after pre-treatment at 4, 25, 30, or 40°C. The optimal pH was determined by incubating the enzyme at pH 4.0-11.0. pH stability was determined by pretreating the enzyme in buffers with varying pH (20 mM citrate-phosphate buffer for pH 4.0–7.0, 20 mM NaH_2_PO_4_-Na_2_HPO_4_ buffer for pH 6.5–9.0, and 20 mM Na_2_CO_3_-NaHCO_3_ buffer for pH 9.0–11.0) at 30°C for 1 h. The residual activity was finally measured after adjusting the pH back to 8.0. Impacts of metal ions and chemicals were also investigated by, respectively, adding them to the reaction mixture at a final concentration of 1 mM. Particularly, the effect of NaCl on enzyme activity was examined by carrying out the reaction at different salt concentrations of 0–0.5 M.

The kinetic parameters of the enzyme were determined in the presence of 0.1–2.0 mg/mL Ib-FUC. Substrate concentrations and enzyme activities were plotted according to the Michaelis-Menten equation using the software GraphPad Prism (GraphPad Software, San Diego, CA, United States), where *K*_*m*_, *V*_*max*_, *K_cat_*, and *K*_*cat*_/*K*_*m*_ were subsequently calculated.

### UPSEC-MS Analysis

To identify the composition of the reaction products, the products were analyzed using UPSEC-MS. The system consisted of an ultrahigh performance liquid chromatography (UPLC) unit (Agilent 1290, Agilent Technologies, Santa Cruz, CA, United States), an ultra-performance size exclusion column (ACQUITY UPLC Protein BEH SEC 125Å Column, 4.6 mm × 300 mm, Waters, Milford, MA, United States), and a quadrupole/time of flight mass spectrometer (Agilent 6545 LC/Q-TOF, Agilent Technologies, Santa Cruz, CA, United States) with the electrospray ionization source. The eluent was 20% (v/v) methanol containing 10 mM ammonium acetate with a flow rate of 0.2 mL/min. Parameters for the mass spectrometer were the gas temperature of 325°C, gas flow at 8 L/min, nebulizer at 40 psi, sheath gas temperature of 250°C, sheath gas flow at 11 L/min, capillary voltage set at 3500 V, the fragmentor at 180 V; skimmer at 45 V and the scanned mass was 200–1200 Da with a negative ion polarity.

### NMR Investigation

The end product was separated on a HiLoad 26/600 Superdex 30 pg column (GE healthcare, Uppsala, Sweden) by using ÄKTA^TM^ Prime Plus system (GE healthcare, Uppsala, Sweden), and eluted with 5 mM ammonium formate (pH 7.0) at a flow rate of 2.6 mL/min. The purified fractions were co-evaporated with D_2_O twice by lyophilization and consequently dissolved in 500 μL D_2_O. The external standard was calibrated according to the eternal 4, 4-dimethyl-4-silapentane-1-sulfonic acid (DSS) (0.00 ppm). The 1-dimensional ^1^H NMR spectrum and 2-dimensional ^1^H−^1^H NMR spectra, including the correlation spectrum (COSY), total correlation spectrum (TOCSY) and nuclear overhauser effect spectroscopy (NOESY) were determined by a Bruker AVANCE III 600 HD spectrometer (Bruker, German) at 600 MHz under 25°C with sufficient acquisition time.

### Transglycosylation Experiments

Transglycosylation related changes were verified using glycerin, methanol,L-fucose, D-glucose, D-galactose, D-fructose, D-mannose, D-glucosamine or N-acetyl-D-glucosamine as acceptors. Different acceptors (2 mg for solid or 0.20 mL for liquid) with 2 mg Ib-FUC were incubated with 0.02 U enzyme in 1 mL 20 mM NaH_2_PO_4_-Na_2_HPO_4_ buffer (pH 8.0) at 25°C for 24 h, which were subsequently analyzed in a UPSEC-MS/MS to detect the generation of glycosylated products. The collision energy to dissociate oligosaccharides in the tandem mass spectrum was set at 45 V, and other parameters were as those described in section “UPSEC-MS Analysis.”

### Bioinformatics Analysis

The homologs of putative endo-1,3-fucanase sequences in the NCBI non-redundant protein sequence database were searched by BLASTP with a cut-off expected threshold value of 1 × 10^–10^ (Altschul et al., 1997). Previously defined glycoside hydrolase domains were predicted by dbCAN (Yin et al., 2012). Multiple sequence alignments were performed using ClustalX (Thompson et al., 1997), and the amino acid sequence alignment was produced using the online application WebLogo server (Crooks et al., 2004). A phylogenetic tree was constructed in MEGA6 (Tamura et al., 2013) using the neighbor-joining algorithm, and tree visualization end editing was performed using Figtree version 1.4.4 (FigTree, 2018).

### Statistical Analysis

All experiments were performed at least three times. The SPSS v11.5 (SPSS Inc., Chicago, IL, United States) was utilized to perform Tukey’s *post hoc* test (ANOVA). A probability value of *p*-value < 0.05 was considered statistically significant.

## Results and Discussion

### Confirmation of Endo-1,3-Fucanase Gene Sequence

#### Discovery of an Endo-1,3-Fucanase Gene Sequence

There were significant differences in gene expression profiles when *W. fucanilytica* was cultured in different media (Supplementary Figure S1). Transcriptomics and differential expression analysis revealed 441 genes up-regulated when grown on the minimal medium containing Ib-FUC compared with the minimal medium containing D-glucose. Among these, 25 genes had expressions higher than 2^6^-fold. Since no endo-1,3-fucanase gene has been identified, we focused on 11 genes that had no prior defined GH domains. These sequences were cloned and heterologously expressed in *E. coli*, five sequences were successfully expressed in the supernatant of cell lysate, and one showed apparent activity on sulfated fucan. This active protein, designated as FunA, was encoded by an open reading frame AXE80_RS10075 (located from 2492018 to 2493244 in the complementary strand, GenBank accession number WP_068826898.1), and composed of 408 amino acid residues containing a putative N-terminal signal peptide (residues M1 to S19). FunA was effectively purified using nickel affinity chromatography (an activity yield of 40.1%). It showed as a single band on SDS-PAGE (Supplementary Figure S3) and an apparent molecular weight (*M*_*w*_) of 48.0 kDa which is consistent with the expected value (48.2 kDa). Type I sulfated fucan Ib-FUC was used to assay the activity due to its regular and clear primary structure (Supplementary Figure S2). The activity of the purified enzyme on Ib-FUC was 13.7 U/mg.

#### Action Mode of FunA Revealed by HPSEC-RID

**FIGURE 1:**
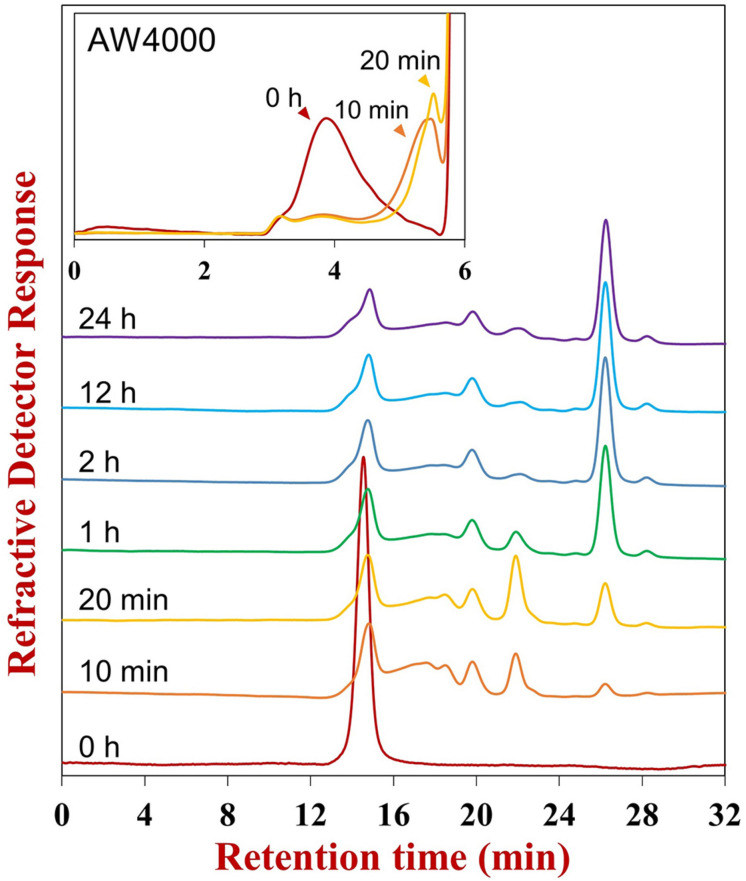
HPSEC-RID chromatograms of hydrolysis products obtained at different reaction times. The reaction time was labeled above the corresponding chromatogram. After 12 h reaction, the fresh enzyme was supplemented into the reaction mixture and incubated for a further 12 h to prepare the end product (24 h). The Superdex peptide 10/300 GL column was employed for examining the oligosaccharide pattern, and the TSKgel SuperAW4000 column was utilized for investigating the global profile (the insert).

In the initial hydrolysis stage, the *M*_*w*_ of Ib-FUC sharply decreased (0–20 min, insert of Figure 1). Meanwhile, the polydispersity of the products increased. Several oligosaccharides were also observed on the HPSEC-RID chromatogram (0–10 min, Figure 1), confirming that FunA was a random endo-acting enzyme. Based on the structure of Ib-FUC (Supplementary Figure S2), it could be inferred that FunA was capable of cleaving the α-1,3 glycosidic linkages within the sulfated fucan in an endo-acting manner. FunA was then further examined as a candidate endo-1,3-fucanase.

FunA was incompetent to hydrolyze sulfated fucans from *F. vesiculosus*, *A. nodosum* and *M. pyrifera* (Supplementary Table S2), which were the effective substrates of GH107 enzymes. Currently, the GH107 family is the only reported fucanase GH family, and its enzymes all exhibited endo-1,4-fucanase activity: MfFcnA was specifically active on α-1,4 glycoside bonds in type II sulfated fucans from *F. vesiculosus* and *A. nodosum* (the primary structures were shown in Supplementary Figure S2; Schultz-Johansen et al., 2018); Fp277 and Fp279 could cleave α-1,4 glycoside bonds in *A. nodosum* (Schultz-Johansen et al., 2018); P5AFcnA and P19DFcnA showed activity on sulfated fucan from *M. pyrifera* (Vickers et al., 2018), though the primary structure of *M. pyrifera* was poorly defined. It has not been reported yet whether the current GH107 enzymes could act on α-1,3 glycosidic linkages. These results demonstrate that FunA was distinct from GH107 enzymes.

### Enzymatic Characterization of FunA

FunA was most active at 40°C and pH 8.0 (Supplementary Figure S4). It could keep stable at 4 and 25°C after 24 h storage, and retain more than 80% of its original activity after exposure to pH 6.5 to 10.0 for 1 h (Supplementary Figure S4). Further, 94.12% of residual activity could be retained after 90 days storage at 4°C without any protective additives, supporting that it was stable. The effects of metal ions on enzyme activity were summarized in Supplementary Table S3. FunA activity was not significantly influenced by NaCl (Supplementary Figure S4E). The activities of marine polysaccharide enzymes were widely reported to depend on the concentration of NaCl, including Cgi82A (Shen et al., 2017) and Por16A_Wf (Zhang Y. et al., 2019) from *W. fucanilytica*. The salt-independent property of FunA would be beneficial to its practical applications. The *K*_*m*_, *V*_*max*_, *K_cat_*, and *K_cat_/K_m_* values of FunA were determined as 3.26 ± 0.30 μM (1.05 ± 0.10 mg/mL), 25.45 ± 0.97 U/mg, 20.46 s^–1^ and 6.28 mM^–1^ s^–1^, respectively.

### Hydrolysis Patterns of FunA

#### Identification of Reaction Products Using UPSEC-MS

**FIGURE 2:**
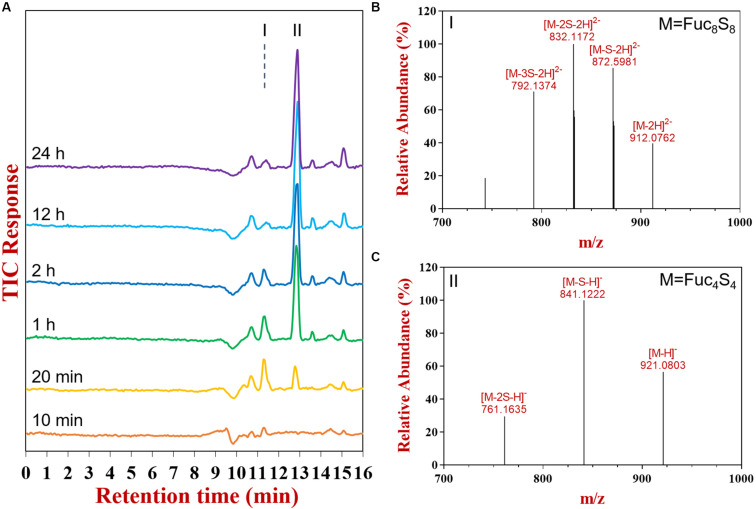
The total ion chromatogram **(A)**, mass spectrums of component I **(B)** and II **(C)** as determined by UPSEC-MS.

The products at different reaction times were identified by liquid chromatograph-mass spectrometry (LC-MS) (Figure 2). The UPSEC was chosen due to its higher separation resolution and better compatibility with mass spectrometry compared with HPSEC. Moreover, UPSEC-MS can achieve the high-throughput detection of the entire glycome and provide abundant structural information. Along with the progress of the enzymatic hydrolysis, two components significantly changed based on total ion chromatograms, which were noted as components I and II (Figure 2A). Component I exhibited an ion peak at m/z 912.0762 (Figure 2B) as [M-H]^2–^ indicating a composition of octasaccharide decorated with eight sulfate groups (Fuc_8_S_8_, 1826.1284 Da). Meanwhile, component II exhibited an ion peak at m/z 921.0803 (Figure 2C) as [M-H]^–^, which was a tetrasaccharide decorated with four sulfate groups (Fuc_4_S_4_, 922.0695 Da).

UPSEC-MS further showed that the octasaccharide (Fuc_8_S_8_) increased in the initial stage of the hydrolysis (0–20 min, Supplementary Figure 2A) followed by a decrease, demonstrating the capability of FunA to cleave it. The tetrasaccharide (Fuc_4_S_4_) consistently accumulated during the reaction. The end product was obtained after 12 h reaction since the oligosaccharide pattern remained unchanged even after further hydrolysis using the excess enzyme and prolonged incubation time (Figures 1, 2A). Quantitative estimation based on the HPSEC-RID spectrum peak area revealed that the yield of tetrasaccharide against all oligosaccharides products was 87.2%, indicating that the major end product of FunA hydrolysis was tetrasaccharide (Fuc_4_S_4_).

#### Structure of the Major Component in the End Product

**FIGURE 3:**
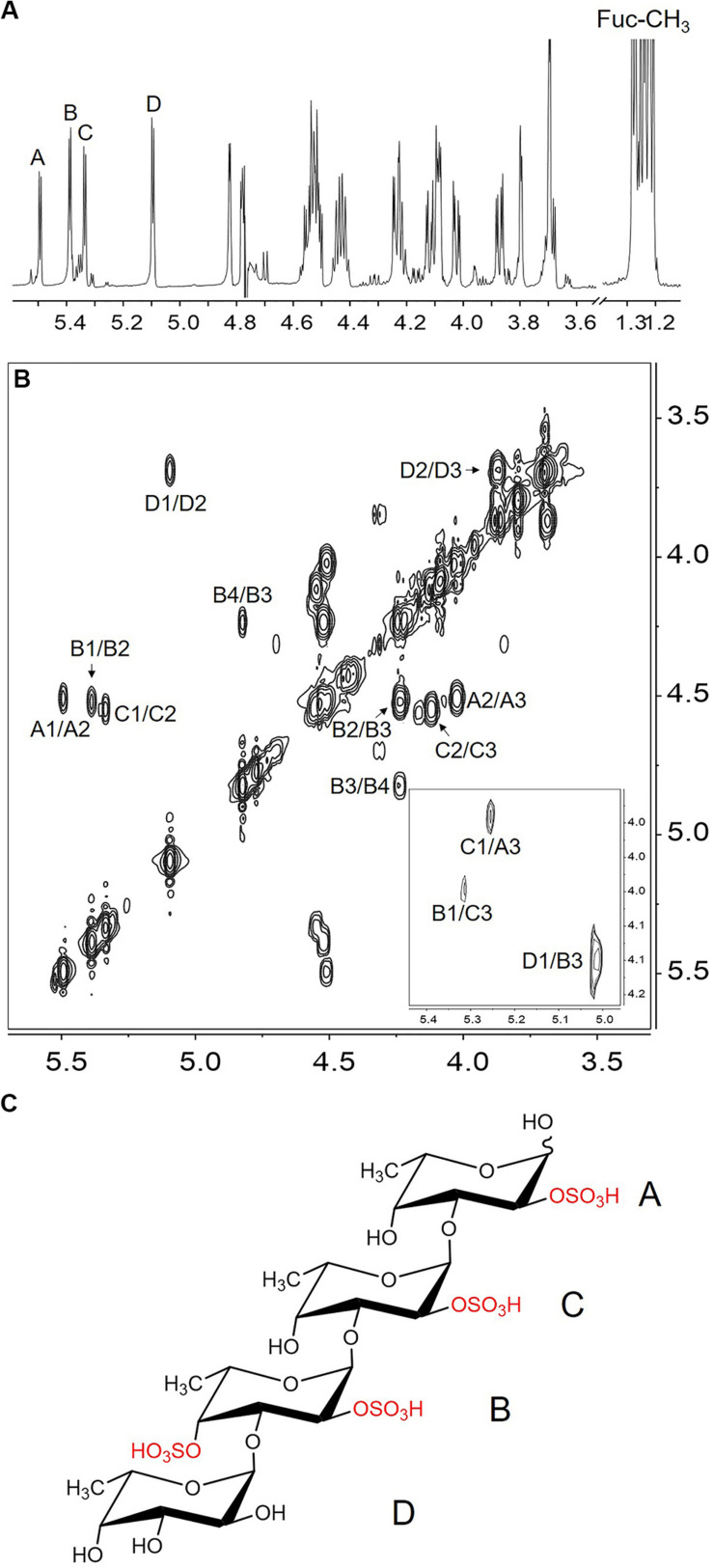
The ^1^H NMR **(A)**, COSY **(B)**, NOESY (insert of B) spectrum and the structure **(C)** of the major component in the end product of FunA. A1/A2 indicated the cross-peak between H-1 and H-2 of residue A, etc.

The major component of the end product was purified and analyzed by NMR. Four spin systems were found in the COSY spectrum (Figure 3B), and their initiative signals were at δ 5.50, δ 5.39, δ 5.34, and δ 5.10 ppm, which could be attributed to the α-anomeric resonance of four fucopyranoside residues designated as A, B, C, and D (Figure 3A). The other proton chemical shifts (Table 1) were assigned according to their correlation peaks in COSY (Figure 3B) and TOCSY (Supplementary Figure S5) spectra. The positions of sulfated substitution could be elucidated by comparing each proton chemical shift with the corresponding proton chemical shift of the non-sulfated fucose residue (Yu et al., 2013). The O-sulfation would cause the chemical shift of oxymethine protons to downfield by 0.4–0.8 ppm (Yamada et al., 1992). Compared with the proton chemical shifts of non-sulfated fucose residue, residues A, B, C, and D were deduced as 2-O-desulfated, 2,4-O-desulfated, 2-O-desulfated, and non-sulfated α-L-fucose residues, respectively. The glycosidic linkage and the sequence of residues were then validated according to the correlation peaks in NOESY spectrum (insert of Figure 3B). Specifically, H-1 of residue D correlated with H-3 of residue B, H-1 of residue B with H-3 of residue C, and H-1 of residue C to H-3 of residue A. The sequence of residues in the tetrasaccharide was consequently confirmed as D1→3B1→3C1→3A. In result, the major end product was determined to be α-L-Fuc*p*-1→3-α-L-Fuc*p*(2,4OSO_3_^–^)-1→3-α-L-Fuc*p*(2OSO_3_^–^)-1→3-α-L-Fuc*p*(2OSO_3_^–^) (Figure 3C).

**TABLE 1:** ^1^H chemical shifts of Ib-FUC tetrasaccharide generated by FunA.

**Residue**	**δH1**	**δH2**	**δH3**	**δH4**	**δH5**	**δH6**
A	5.50	**4.51**^a^	4.02	4.08	4.09	1.23
B	5.39	**4.52**	4.24	**4.82**	–^c^	1.28
C	5.34	**4.55**	4.12	4.09	4.11	1.28
D	5.10	3.69	3.87	4.07	–	1.25
→3-α-L-Fuc*p*-1→^b^	5.07	3.83	3.93	4.02	4.27	1.25

*^*a*^ The chemical shifts highlighted in bold were down-field shifted compared with the corresponding ^1^H chemical shifts of non-sulfated α-L-Fuc*p*. The chemical shifts were expressed in ppm. ^1^H chemical shifts were referred to as 4,4-dimethyl-4-silapentane-1-sulfonic acid (δ 0.00 ppm). ^*b*^ The chemical shifts of →3-α-L-Fuc*p*-1→ was referred to as the previous report (Chang et al., 2016). ^*c*^ “–”: not detected.*

#### Investigation on Cleavage Point

Compared to the structure of Ib-FUC polysaccharide (Supplementary Figure S2), it could be concluded that FunA cleaved the α-1,3-glycoside bonds between 2-O-sulfated and non-sulfated fucose residues in Ib-FUC (Supplementary Figure S6). Besides, FunA was incompetent to hydrolyze sulfated fucans containing α-1,4-glycoside bonds based on the result mentioned above (Supplementary Table S2). Those results further confirmed that FunA is an endo-1,3-fucanase. It should be noted that FunA could not recognize other α-1,3-glycoside bonds in Ib-FUC such as the glycosidic linkages between non-sulfated and 2,4-O-sulfated, between 4-O-sulfated and 2-O-sulfated, or between 2-O-sulfated and 2-O-sulfated fucose residues. It is well known that glycoside hydrolase is a class of enzyme with high specificity to glycosidic linkage and structural modifications. A good example is carrageenase. The κ- and ι-carrageenan share the identical structure except for the additional sulfation on the O-2 position of the anhydro-galactose residue of ι-carrageenan. Nevertheless, their hydrolysis should be implemented by enzymes belonging to two distinctive glycoside hydrolase families, viz., GH16 κ-carrageenase for κ-carrageenan and GH82 ι-carrageenase for ι-carrageenan, demonstrating strong specificity of the enzymes to the sulfated pattern (Matard-Mann et al., 2017). Similarly, the specificity of fucanases might also involve the specific recognition for the sulfated pattern of the substrate. Besides, as shown in section “Confirmation of Endo-1,3-Fucanase Gene Sequence”, FunA was inactive to type II sulfated fucans, confirming that it was incapable of cleaving the α-1,3 glycosidic linkages within the backbone composed of alternating 1,3- and 1,4-linked α-L-fucopyranose residues. FunA has strict specificity, implying that this enzyme could be utilized as a biotechnological tool in the structural investigation of type I sulfated fucans. Besides, FunA could also promote the application of endo-1,3-fucanase in bio-industry such as specific production of α-1,3-linked sulfated fucan oligosaccharides.

### The Transglycosylating Activity of FunA

**FIGURE 4:**
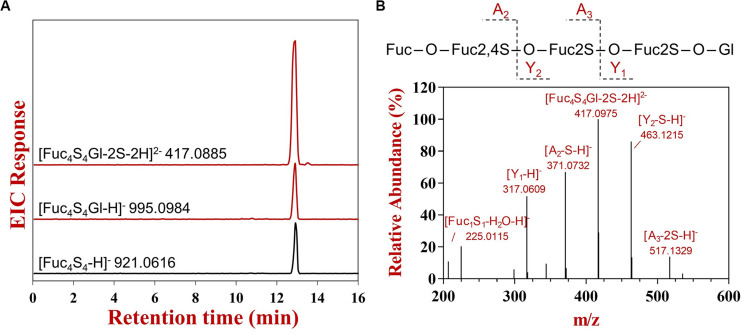
The UPSEC-MS analysis of hydrolysis and transglycosylating products produced by FunA in the reaction system with glycerin as the acceptor. **(A)** Extraction ion chromatograms; **(B)** MS/MS spectrum of [Fuc_4_S_4_Gl-2S-2H]^2–^. “Fuc” represented fucose residue, “Gl” represented glycerin residue, “S” represented sulfate group, “O” represented oxygen from the glycosidic bond. The charge number of fragment was provided by the instrumental software (Agilent Masshunter Qualitative Analysis Navigator B.08.00).

The glycoside hydrolases with transglycosylating activity were regarded as the potential biocatalysts for the large-scale synthesis of oligosaccharides or complex carbohydrates (Raich et al., 2016). To confirm the transglycosylating activity for FunA, different acceptors were tested in this experiment. The extracted ion chromatograms of all expected glycosylated products with or without desulfation were thoroughly inspected. No transglycosylating products were observed in sample absented acceptors and the reactions with D-glucose, D-galactose, D-fructose, D-mannose, D-glucosamine, and N-acetyl-D-glucosamine as the acceptor under the conditions of the present study, while the expected ions were detected in the reactions with glycerin, methanol, and L-fucose (Figure 4A and Supplementary Figure S7). To further confirm transglycosylation, the generated products with glycerin as acceptor were detected by UPSEC-MS/MS. The ion [Fuc_4_S_4_Gl-2S-2H]^2–^ (m/z 417.0885, Figure 4A) was selected and analyzed in MS/MS due to its high intensity in the MS spectrum. As shown in Figure 4B, critical fragments (Y_1_ and Y_2_) containing glycerin aglycone were confirmatively detected in MS/MS, indicating that glycerin was linked to Ib-FUC tetrasaccharide. The results further confirmed FunA is capable to catalyze transglycosylation.

The glycoside hydrolases generally act via retaining or inverting mechanisms (Mccarter and Withers, 1994). Previous studies showed that glycoside hydrolases with transglycosylating activity were all adopted retaining mechanism, which ascribed to the two steps catalytic mechanism in retaining glycoside hydrolases: glycosylation and deglycosylation. The transglycosylated product is generated when the acceptor was a molecule with hydroxyl groups instead of water in the deglycosylation reaction (Mccarter and Withers, 1994). To the best of our knowledge, no inverting GHs were reported to possess transglycosylating activities. The transglycosylating activity of FunA implied its retaining mechanism in catalysis. This reaction mechanism is in general determined by NMR measurements (Eneyskaya et al., 2001), while the attempt to monitor the changes of anomeric configurations in the reaction by using ^1^H NMR failed since chemical shifts for β-α-L-Fucp(2OSO_3_^–^) could not be figured out.

### Proposal for a Novel GH Family Containing Endo-1,3-fucanase

#### Bioinformatics Analysis and Proposal of a Novel GH Family Containing Endo-1,3-Fucanase

**FIGURE 5:**
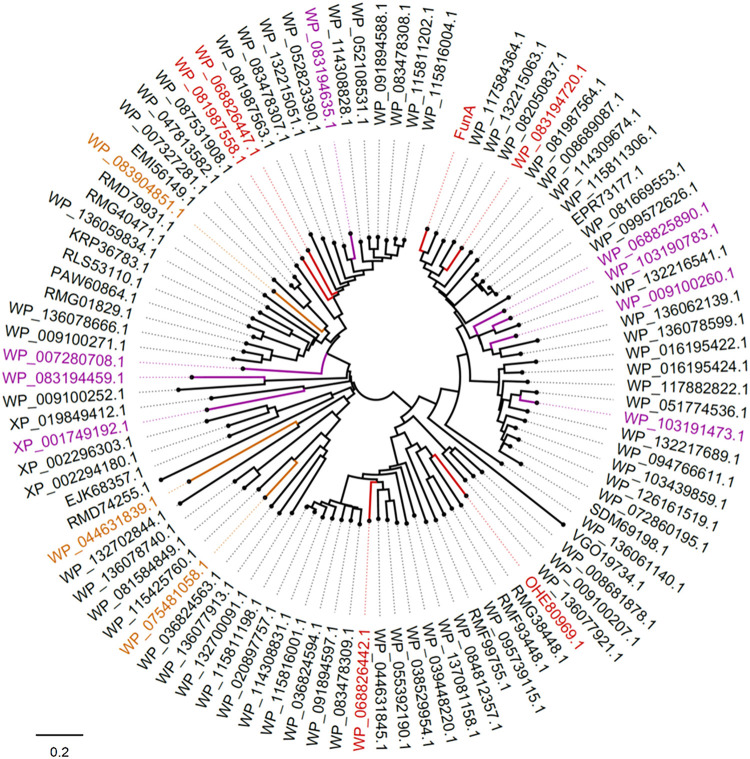
Phylogenetic analysis of FunA and its homologs. Sequences successfully expressed in the supernatant of *E. coli* cell lysate were highlighted: sequences exhibited activity on Ib-FUC were in red; sequences with no activity on Ib-FUC were in purple; sequences expressed in inclusion body were in yellow.

Using the BLASTP algorithm, 97 potential homologs of FunA were found in the NCBI database using FunA as the query sequence. They were originated from seven phyla of Bacteria (Bacteroidetes, Proteobacteria, Planctomycetes, Kiritimatiellaeota, Lentisphaerae, Verrucomicrobia, and Sphingobacteriia) and three phylum of Eukaryota (Stramenopiles, Choanoflagellida, and Metazoa). None of the previously reported glycoside hydrolase domains were predicted in all of the sequences analyzed. And those potential homologs of FunA were no significant similarity with enzymes of GH107 family according to BLASTP results. The 16 representative sequences, which had distinct clustering in the phylogenetic tree (Figure 5) were further synthesized, cloned, expressed and characterized. The synthesis of candidate α-1,3-fucanase genes was entrusted to Sangon Biotech (Shanghai) Co., Ltd (Shanghai, China), where 13 out of the 16 sequences were successfully expressed in the supernatants of cell lysate (Figure 5). Five proteins showed apparent activity to Ib-FUC, namely WP_081987558.1, WP_068826447.1, WP_068826442.1, OHE80969.1, and WP_083194720.1 (activities were listed in Supplementary Table S4). The sharp decreases of substrate *M*_*w*_ in the initial hydrolysis stage indicated that these five sequences all hydrolyzed Ib-FUC in an endo-acting manner (Supplementary Figure S8). The novelty of FunA and its homologs in sequence and in activity suggests the existence of a novel fucanase family (assigned as the family GH168 by CAZy).

It should also be noticed that eight sequences (Figure 5, highlighted in purple; Supplementary Figure S9) were successfully expressed while inactive to sulfated fucan substrates employed in the current study. It is well known that many glycoside hydrolase families comprise multiple subfamilies with various substrate specificities, such as GH5, GH16, GH29, and GH95. We supposed that these eight sequences might be specific to other forms of the substrates, warranting further research on the diversity of enzymes in the newly proposed GH family.

#### Identification of Critical Sites of FunA

Multiple sequence alignment of FunA and its homologs showed that D206 and E264 in FunA were strictly conserved in all sequences (Supplementary Figure S10). Generally, the catalytic residues in GH families are acidic amino acids Asp or Glu. To verify the functions of D206 and E264 in FunA, two single-site mutants D206E and E264Q were established and successfully expressed in *E. coli* (Supplementary Figure S11), and then purified by HisTrap HP columns (Supplementary Figure S11). The purified D206E and E264Q were all inactive on Ib-FUC, indicating that D206 and E264 were critical for the activity of FunA.

## Conclusion

The first endo-1,3-fucanase gene funA was found via transcriptomics from marine bacterium *W. fucanilytica* CZ1127^T^. The enzymatic properties and hydrolysis patterns of FunA were further characterized. FunA specifically catalyzed α-1,3 glycosidic linkage between 2-O-sulfated and non-sulfated fucose residues in a random endo-acting manner. It exhibited transglycosylating activities with glycerine, methanol, and L-fucose as acceptors, implying a possible retaining hydrolysis mechanism of FunA. D206 and E264 were critical for the activity of FunA. The endo-1,3-fucanase activity of the homologs of FunA was also verified. The discovery and characterization of FunA proposed a novel GH family (GH168) containing endo-1,3-fucanase.

## Data Availability Statement

The datasets presented in this study can be found in online repositories. The names of the repository/repositories and accession number(s) can be found at: https://www.ncbi.nlm.nih.gov/genbank/, WP_068826898.1.

## Author Contributions

JS and YC conceptualized and designed the studies. JS, YZ, and XM performed the experimental operations. JS performed the data analyses. JS and YC wrote the manuscript. YC and CX revised the manuscript. All the authors read and approved the final manuscript.

## Conflict of Interest

The authors declare that the research was conducted in the absence of any commercial or financial relationships that could be construed as a potential conflict of interest.

## Footnotes

1 http://www.cazy.org

## Supplementary Material

FIGURE S1 The volcano plot of differentially expressed genes. The red dots represent the up-regulated genes of *W. funcanilytica* induced by growth on the sulfated fucans culture medium, and the green dots represent the down-regulated genes.

FIGURE S2 Structures of sulfated fucans from sea cucumber *I. badionotus (Ib)*, algae *F. vesiculosus (Fv)* and algae *A. nodosum (An)*.

FIGURE S3 SDS-PAGE of the purified recombinant FunA.

FIGURE S4 Biochemical properties of FunA: (A) optimal temperature; (B) thermal stability; (C) optimal pH; (D) pH stability; (E) influence of NaCl concentration.

FIGURE S5 The TOCSY spectrum of the major component in the end product of FunA. A1/A2 indicated the cross-peak between H-1 and H-2 of residue A, etc.

FIGURE S6 The cleavage point of FunA acting on Ib-FUC. Arrows represented the cleavage point.

FIGURE S7 Extracted ion chromatograms of the transglycosylating products with methanol (A) and fucose (B) as acceptors. “Me” represented the methyl group. The ions of corresponding canonical hydrolysis products were shown as references for a comparison of abundance.

FIGURE S8 Global profiles of the reaction progress in the initial hydrolysis stage of WP_081987558.1 **(A)**, WP_068826447.1 **(B)**, WP_068826442.1 **(C)**, OHE80969.1 **(D)** and WP_083194720.1 **(E)**. Ib-FUC was used as the substrate. The TSKgel SuperAW4000 column was employed to investigate the global profile.

FIGURE S9 SDS-PAGE analysis of recombinant potential homologues of FunA. M: *M_w_* standard markers; Line 1: control group; Line 2: cell lysate supernatant of WP_103190783.1; Line 3: cell lysate supernatant of WP_103191473.1; Line 4: cell lysate supernatant of WP_007280708.1; Line 5: cell lysate supernatant of XP_001749192.1; Line 6: control group; Line 7: cell lysate supernatant of WP_083194459.1; Line 8: cell lysate supernatant of WP_009100260.1; Line 9: cell lysate supernatant of WP_068825890.1; Line 10: cell lysate supernatant of WP_083194635.1; Line 11: control group. The over-expressed proteins were indicated with red rectangles.

FIGURE S10 Multiple sequence alignment of residues in FunA and its homologues. The strictly conserved residues in all sequences were indicated with black triangles.

FIGURE S11 SDS-PAGE analysis of the mutants. M: *M*_*w*_ standard markers; Line 1: control group; Line 2: cell lysate supernatant of mutant D206E; Line 3: cell lysate supernatant of mutant E264Q; Line 4: the purified D206E; Line 5: the purified E264Q. The over-expressed proteins in cell lysate supernatants were indicated with red rectangles.

TABLE S1 The forward and reverse primers of FunA and its mutants.

TABLE S2 The enzymatic activities of FunA hydrolyzed sulfated fucans from brown algae.

TABLE S3 Effect of metal ions and chemicals on the activity of FunA.

TABLE S4 Activities of endo-1,3-fucanases acted on sulfated fucan from *I. badionotus*.
