# Supplementary material for: Discovery and Characterization of an Endo-1,3-Fucanase From Marine Bacterium Wenyingzhuangia fucanilytica: A Novel Glycoside Hydrolase Family
Source: Front Microbiol. 2020 Jul 28;11:1674. doi: 10.3389/fmicb.2020.01674 (PMC7401878; doi:10.3389/fmicb.2020.01674)
Supplement: FIGURE S1 — The volcano plot of differentially expressed genes. The red dots represent the up-regulated genes of W. funcanilytica induced by growth on the sulfated fucans culture medium, and the green dots represent the down-regulated genes. [file Data_Sheet_1.pdf]

Supplementary Material

1 Supplementary Figures

1.1 Figure S1

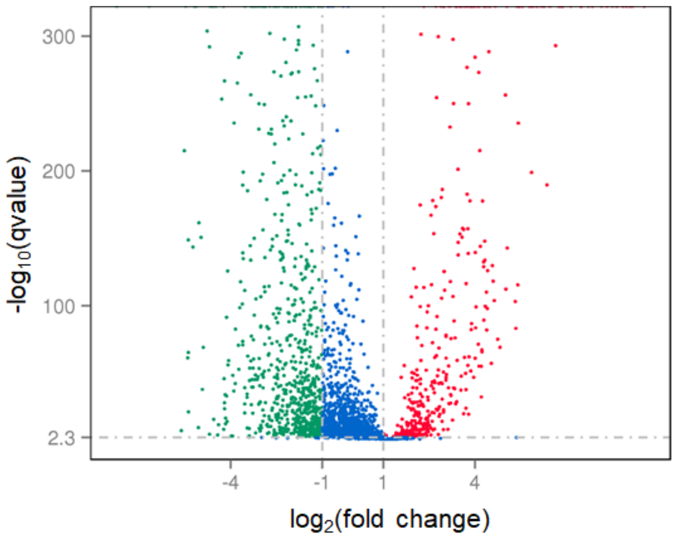

Supplementary Figure 1. The volcano plot of differentially expressed genes. The red dots represent the up-regulated genes of *W. funcanilytica* induced by growth on the sulfated fucans culture medium, and the green dots represent the down-regulated genes.

1.2 Figure S2

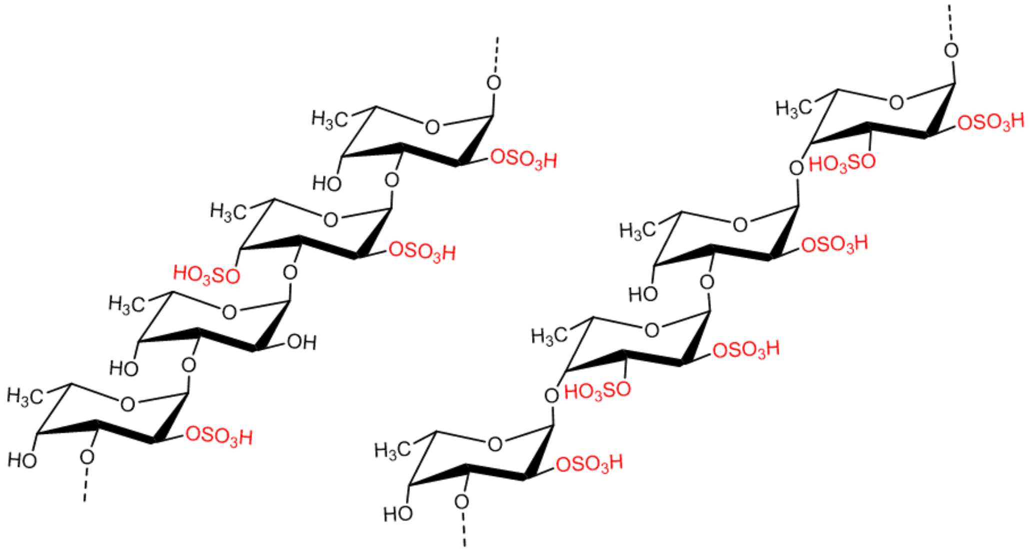

Ib *Fv* , *An*

Supplementary Figure 2. Structures of sulfated fucans from sea cucumber *I. badionotus* (*Ib*) (Chen et al., 2012), algae *F. vesiculosus* (*Fv*) (Ale & Meyer, 2013) and algae *A. nodosum* (Kusaykin, Silchenko, Zakharenko & Zvyagintseva, 2016).

1.3 Figure S3

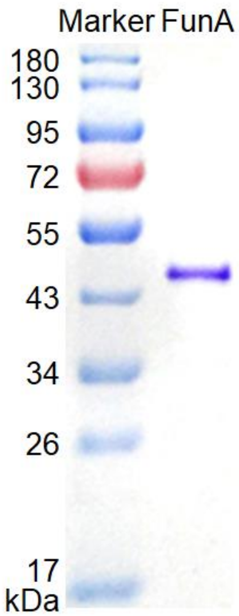

Supplementary Figure 3. SDS-PAGE of the purified recombinant FunA.

1.4 Figure S4

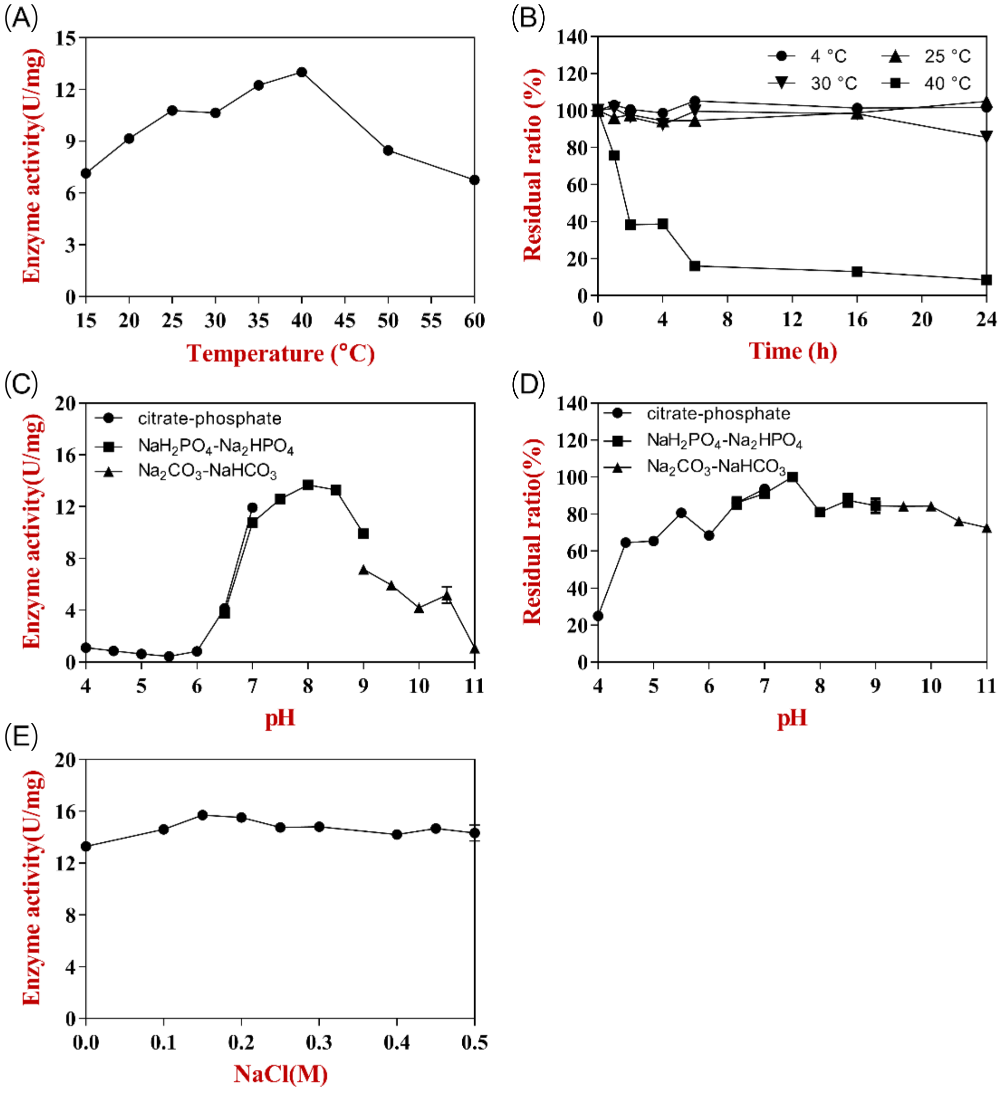

Supplementary Figure 4. Biochemical properties of FunA: (A) optimal temperature; (B) thermal stability; (C) optimal pH; (D) pH stability; (E) influence of NaCl concentration.

1.5 Figure S5

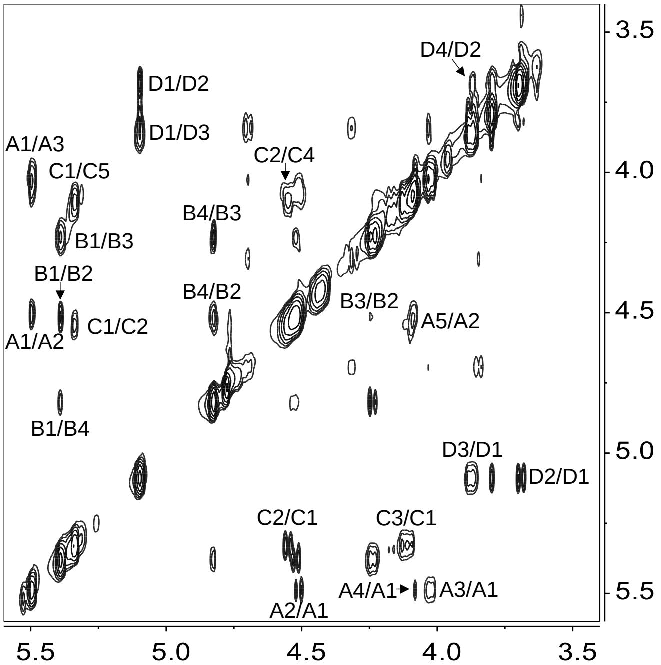

Supplementary Figure 5. The TOCSY spectrum of the major component in the end product of FunA. A1/A2 indicated the cross-peak between H-1 and H-2 of residue A, etc.

1.6 Figure S6

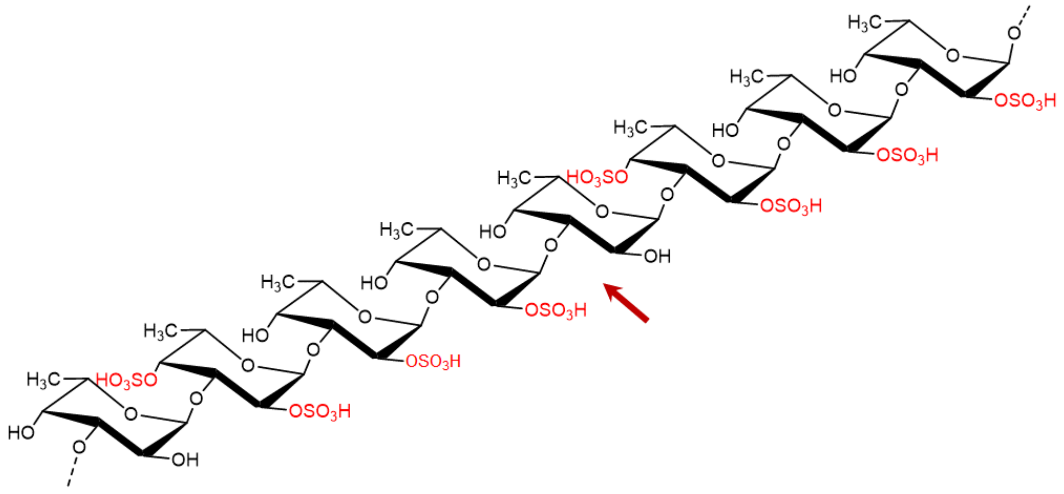

Supplementary Figure 6. The cleavage point of FunA acting on Ib-FUC. Arrows represented the cleavage point.

1.7 Figure S7

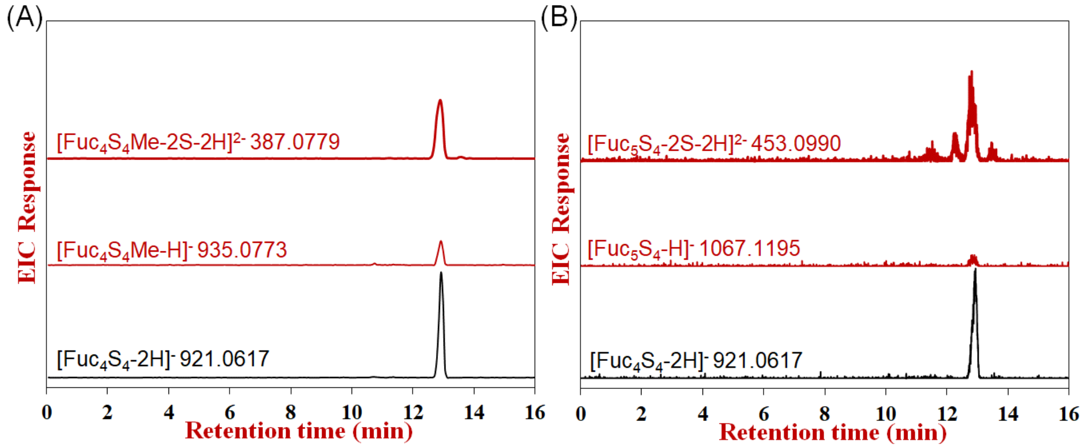

Supplementary Figure 7. Extracted ion chromatograms of the transglycosylating products with methanol (A) and fucose (B) as acceptors. “Me” represented the methyl group. The ions of corresponding canonical hydrolysis products were shown as references for a comparison of abundance.

1.8 Figure S8

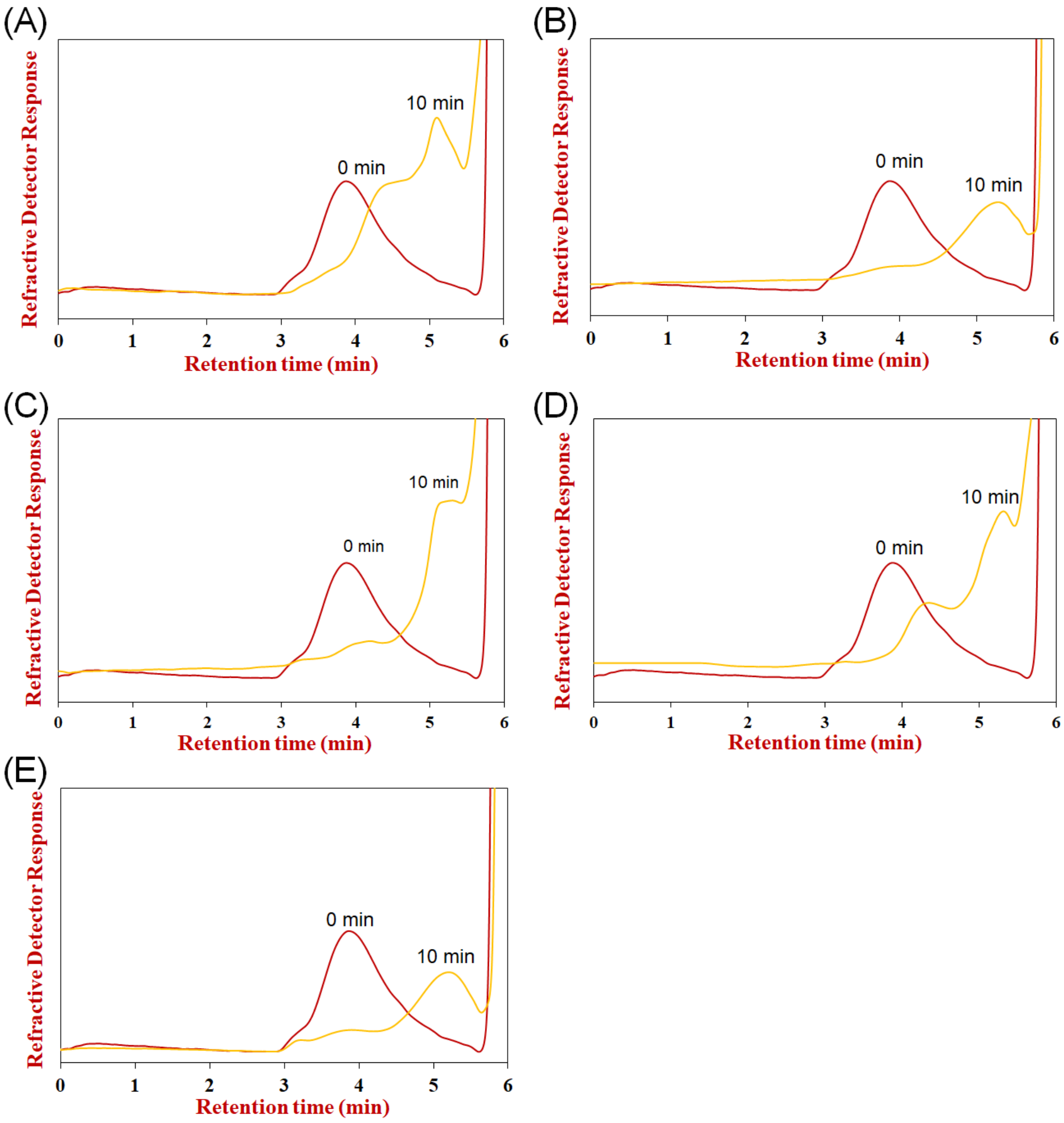

Supplementary Figure 8. Global profiles of the reaction progress in the initial hydrolysis stage of WP_081987558.1 (A), WP_068826447.1 (B), WP_068826442.1 (C), OHE80969.1 (D) and WP_083194720.1 (E). Ib-FUC was used as the substrate. The TSKgel SuperAW4000 column was employed to investigate the global profile.

1.9 Figure S9

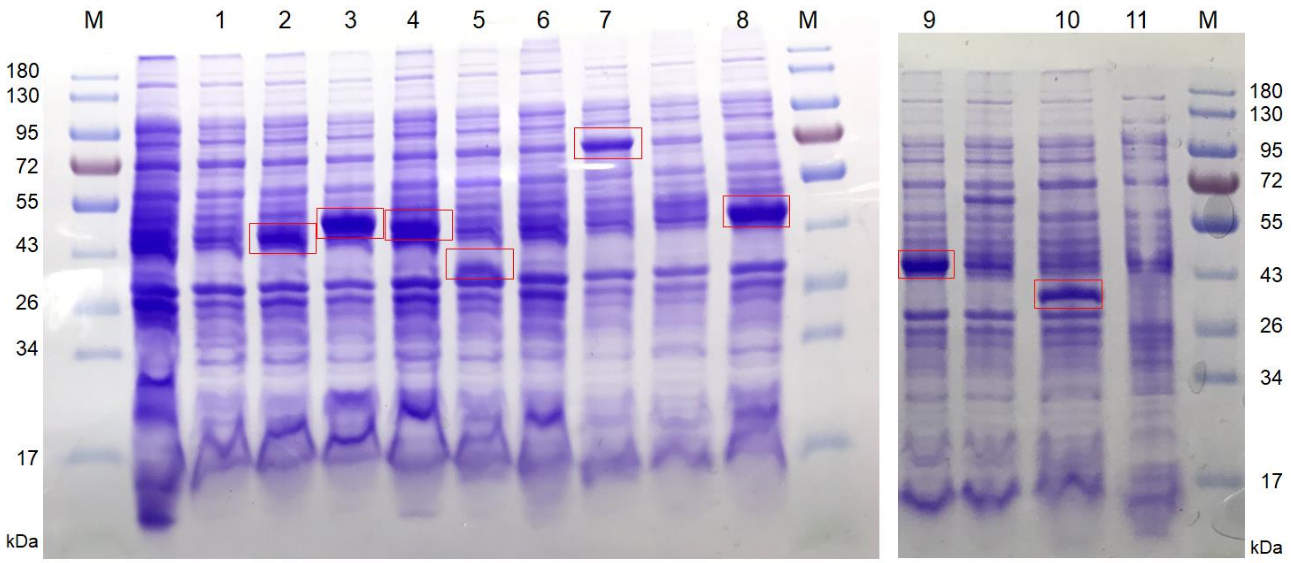

Supplementary Figure 9. SDS-PAGE analysis of recombinant potential homologues of FunA. M: M_w standard markers; Line 1: control group; Line 2: cell lysate supernatant of WP_103190783.1; Line 3: cell lysate supernatant of WP_103191473.1; Line 4: cell lysate supernatant of WP_007280708.1; Line 5: cell lysate supernatant of XP_001749192.1; Line 6: control group; Line 7: cell lysate supernatant of WP_083194459.1; Line 8: cell lysate supernatant of WP_009100260.1; Line 9: cell lysate supernatant of WP_068825890.1; Line 10: cell lysate supernatant of WP_083194635.1; Line 11: control group. The over-expressed proteins were indicated with red rectangles.

1.10 Figure S10

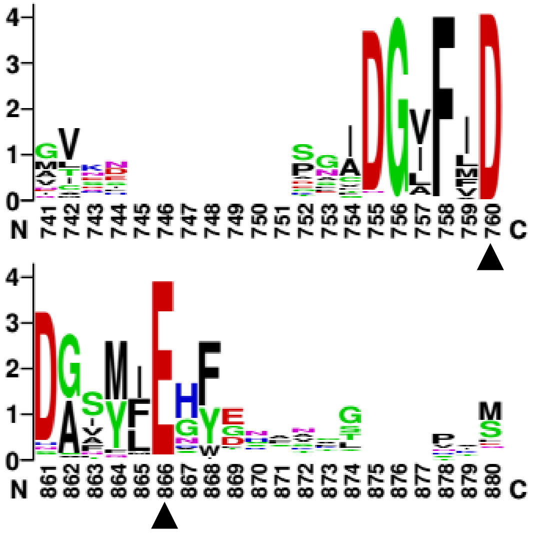

Supplementary Figure 10. Multiple sequence alignment of residues in FunA and its homologues. The strictly conserved residues in all sequences were indicated with black triangles.

1.11 Figure S11

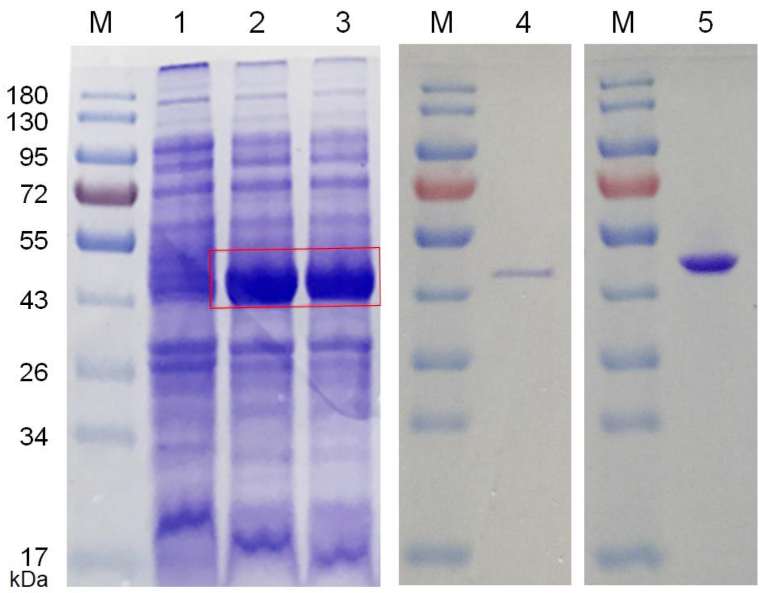

Supplementary Figure 11. SDS-PAGE analysis of the mutants. M: M_w standard markers; Line 1: control group; Line 2: cell lysate supernatant of mutant D206E; Line 3: cell lysate supernatant of mutant E264Q; Line 4: the purified D206E; Line 5: the purified E264Q. The over-expressed proteins in cell lysate supernatants were indicated with red rectangles.

2 Supplementary Tables

2.1 Table S1

Supplementary Table 1. The forward and reverse primers of FunA and its mutants.

Mutants.	Primers 5’-3’
FunA	1+: GACACGGATCCTGTAGTACAACAAAAACACACACCAATAC
	1-: GTGTCCTCGAGTTACTTTTTCCATTCAATTTTCGCTTCT
D206E	1+: CAGCAAATGGGTCGCGGATCCTGTAGTACAACAAAAACACACACC
	1-: GCTCTATAAAAACTCCATCAGCACCAGAGT
	2+: GATGGAGTTTTTATAGAGCAAATGCATGGT
	2-: TCTCAGTGGTGGTGGTGGTGGTGCTCGAGTTACTTTTTCCATTCAATTTTCGC
E264Q	1+: CAGCAAATGGGTCGCGGATCCTGTAGTACAACAAAAACACACACC
	1-: GCTGAAACATAGCTGCATCAATCGCAGG
	2+: GATGCAGCTATGTTTCAGCATTATAATAAT
	2-: TCTCAGTGGTGGTGGTGGTGGTGCTCGAGTTACTTTTTCCATTCAATTTTCGC

2.2 Table S2

Supplementary Table 2. The enzymatic activities of FunA hydrolyzed sulfated fucans from brown algae.

Soure	Enzymatic activity (U/mg)
F. vesiculosus	0.04
A. nodosum	0.11
M. pyrifera	0.08

2.3 Table S3

Supplementary Table 3. Effect of metal ions and chemicals on the activity of FunA.

Compounds	Relative activity (%)	Compounds	Relative activity (%)
None	100	Ba^{2+}	89.29±1.50
Li^{+}	87.59±1.02	Cu^{2+}	-0.63±2.06
K^{+}	94.47±1.08	Pb^{3+}	60.01±0.28
Ni^{2+}	12.25±0.57	Cr^{3+}	69.30±0.16
Mg^{2+}	94.84±0.59	Al^{3+}	61.05±0.43
Hg^{2+}	21.92±0.75	SDS	10.26±1.97
Zn^{2+}	41.63±0.66	$EDTA \cdot Na_2$	81.05±1.50
Mn^{2+}	90.52±1.57	β-mercaptoethanol	81.14±0.59

2.4 Table S4

Supplementary Table 4. Activity of endo-1,3-fucanases acted on sulfated fucan from *I. badionotus*.

Genebank No.	Activitiy (U/mL)
WP_081987558.1	0.38
OHE80969.1	0.59
WP_068826442.1	0.12
WP_068826447.1	3.41
WP_083194720.1	3.89

3 References

Ale, M. T., and Meyer, A. S. (2013). Fucoidans from brown seaweeds: an update on structures, extraction techniques and use of enzymes as tools for structural elucidation. *RSC Advances*, 3, 8131-8141. doi: 10.1039/c3ra23373a

Chen, S., Hu, Y., Ye, X., Li, G., Yu, G., Xue, C., and Chai, W. (2012). Sequence determination and anticoagulant and antithrombotic activities of a novel sulfated fucan isolated from the sea cucumber *Isostichopus badionotus*. *BBA-GEN SUBJECTS*, 1820, 989-1000. doi: 10.1016/j.bbagen.2012.03.002

Kusaykin, M. I., Silchenko, A. S., Zakharenko, A. M., and Zvyagintseva, T. N. (2016). Fucoidanases. *Glycobiology*, 26, 3-12. doi: 10.1093/glycob/cwv072
